# Dual-Targeting Strategy to Repurpose Cetuximab with HFn Nanoconjugates for Immunotherapy of Triple-Negative Breast Cancer

**DOI:** 10.1021/acsami.5c06626

**Published:** 2025-05-06

**Authors:** Linda Barbieri, Lucia Salvioni, Andrea Banfi, Stefania Garbujo, Luisa Fiandra, Chiara Baioni, Marco Giustra, Lucia Morelli, Gianni Frascotti, Miriam Colombo, Metello Innocenti, Davide Prosperi

**Affiliations:** Department of Biotechnology and Biosciences, 9305 University of Milano-Bicocca, Piazza Della Scienza 2, 20126 Milan, Italy

**Keywords:** triple-negative breast cancer, cetuximab, ferritin, nanoconjugates, epidermal growth factor receptor, transferrin receptor, immunotherapy

## Abstract

Triple-negative breast cancer (TNBC) is a highly aggressive and treatment-resistant malignancy characterized by the lack of targeted therapies and poor clinical outcomes. Here, we present a dual-targeting strategy combining the anti-EGFR monoclonal antibody cetuximab (CTX) with H-ferritin (HFn), a nanoparticle targeting transferrin receptor 1 (TfR1), for potential immunotherapy in CTX-resistant tumors. The HFn–CTX nanoconjugate exhibited favorable biophysical properties and good tumor accumulation and significantly enhanced antibody-dependent cellular cytotoxicity (ADCC) in TNBC spheroids compared to CTX alone. Conversely, glioblastoma spheroids did not exhibit comparable reactivity. This effect correlated with elevated cell-surface EGFR expression and plasma-membrane lingering of the nanoconjugate in TNBC cells, facilitating robust immune activation. Biodistribution studies showed selective accumulation of the HFn–CTX nanoconjugate in TNBC tumors in vivo. These findings highlight the potential of HFn–CTX nanoconjugates to repurpose CTX for refractory cancers that express EGFR at high levels, such as TNBC, leveraging dual-receptor targeting to amplify immune-mediated cytotoxicity and overcome resistance.

## Graphical abstract

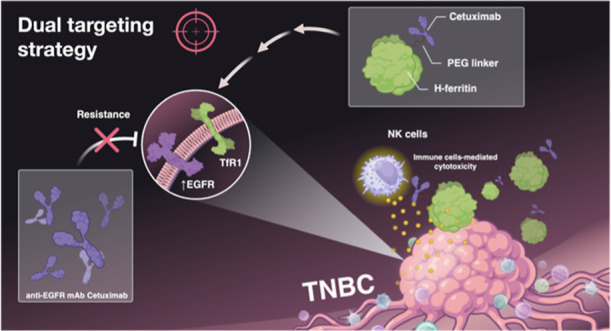

## Introduction

Breast cancer (BC) is the most frequent malignancy in women worldwide and remains the second most common cause of cancer death among females. BC represents about 12% of all new cancer cases each year and 25% of all cancers in women. Early stage noninvasive or locally invasive BC can be cured in almost 70–80% of patients. In contrast, advanced (metastatic) BC remains incurable because the available therapeutic options can only delay progression or serve as palliative treatments.^1^

The breast tumors that do not express estrogen receptor, progesterone receptor, or human epidermal growth factor receptor 2 (HER2, aka ErbB-2) are referred to as triple-negative breast cancer (TNBC), which accounts for approximately 11–20% of all female BC cases and has the highest mortality rate.^2^ In keeping with this, TNBC shows a high histological grade, a high proliferation rate, a high risk of relapse, and short progression-free and overall survival.^3,4^

TNBC treatment is challenging due to the lack of obvious drug targets, with surgery, chemotherapy, and radiation remaining the mainstay of standard treatment.^3,5,6^ Hence, developing effective targeted therapies for TNBC is an urgent medical need. In this regard, it is tantalizing that 40–60% of all TNBC overexpress epidermal growth factor receptor 1 (EGFR, aka HER1, aka ErbB-1), a well-known proto-oncogene.^7,8^

Several EGFR-targeting mAbs have been developed in recent years. Among them, cetuximab (CTX) binds to the extracellular domain of EGFR with high affinity and competes with endogenous ligands, thereby inhibiting EGFR signaling. This produces antitumor effects such as cell-cycle arrest, induction of apoptosis, inhibition of angiogenesis and metastasis, and enhanced sensitivity to radio- or chemotherapy.^9^ Besides anticancer effects mediated by the binding to cell-surface EGFR, CTX can promote immune-cell-induced tumor killing through a process called antibody-dependent cellular cytotoxicity (ADCC). The ADCC induced by CTX is EGFR-dependent and mediated by the activation of natural killer (NK) cells. Notably, these immunomodulatory properties were found to contribute to the antitumor effects observed in the clinic.^10,11^ Yet, the use of CTX as an anti-EGFR therapeutic has only been approved for the treatment of patients with wild-type RAS metastatic colorectal cancer and head and neck squamous cell cancer.

This is partly related to its suboptimal pharmacokinetics and relatively short circulation half-life (4.5 and 1.5 days in humans and mice, respectively) compared to other mAbs, likely due to CTX being a chimeric IgG1, limited tumor penetration, inability to cross biological barriers, and partly to several types of resistance mechanisms.^12–14^ Nanotechnology can help bypass some of these drawbacks, and different types of EGFR-targeted nanoparticles relying on mAbs have shown some effects on TNBC in vitro.^15–17^ Although mAb-based active tumor targeting makes these nanoparticles potentially superior to those counting on the enhanced permeability and retention (EPR) effect for accumulation in the tumor, selectivity remains an issue. Indeed, they would be retained in all bodily tissues expressing the EGFR at appreciable levels.^18^

An elegant approach to enhance the specificity and levels of nanoparticle accumulation within tumors would be combinatorial targeting of tumor surface antigens. A prime example is transferrin receptor 1 (TfR1, aka CD71), a cell-surface antigen overexpressed in 98% of human solid cancers, including TNBC.^19–22^ For this reason, the high-affinity TfR1 ligand H-ferritin (HFn), a fully biocompatible recombinant round-shaped cage protein that can be loaded with various payloads, has attracted much interest in the nanomedicine field. As the HFn nanoparticles exhibited good tumor targeting and the ability to cross the blood brain barrier,^23–26^ HFn has been exploited as a drug delivery system for chemotherapeutics improving their accumulation in the tumor while reducing adverse side effects.^27^ Moreover, the surface of the HFn cage can be modified, both genetically and chemically, to add functional moieties. In particular, surface primary amines were successfully conjugated to peptides, antibodies, or fluorophores.^28,29^

Here, we leverage both the high affinity and specificity of HFn and CTX for their cognate receptors to develop a novel HFn–CTX nanoconjugate with dual targeting and repurpose CTX for the treatment of a wider array of cancers. We set up a tailored pipeline to produce and purify the HFn–CTX nanoconjugate, the uptake of which was assessed in TNBC and glioblastoma (GBM) cells expressing high EGFR levels and harboring K-*ras* or PTEN mutations associated with CTX resistance.^30,31^ To weigh the HFn–CTX nanoconjugate as a valuable tool to kill CTX-resistant cells, we measured the immune-mediated anticancer activity of the nanoconjugate in 3D cultures. Surprisingly, we discovered that its ability to induce ADCC was correlated to the cell-surface EGFR and TfR1 levels and internalization kinetics. Furthermore, the HFn–CTX nanoconjugate showed a favorable biodistribution and good tumor accumulation and retention in a preclinical mouse model of TNBC. Taken together, these results open the door to repurposing CTX with HFn nanoconjugates for immunotherapy of patients with TNBC or other cancers that are ineligible to standard CTX treatments.

## Experimental Section

### Reagents and Antibodies

Mammalian cell culture media and reagents were purchased from Euroclone. If not otherwise specified, all other chemicals were from Sigma-Aldrich.

Primary antibodies: CD44 (clone IM7; Bio-Rad, MCA4703), CD31/PECAM-1 (Biotechne, AF3628), CD71 (clone D7G9X, Cell Signaling, 13113),^32,33^ EGFR (clone D38B1, Cell Signaling, 4267), β-Actin (clone D6A8, Cell Signaling, 8457), EGFR (clone 528, Merck, GR01), CD71 (clone MEM-75, Invitrogen, MA1-19137), and Ferritin Heavy Chain (clone EPR3005Y, Abcam, ab75972).

Secondary antibodies: Alexa Fluor-conjugated secondary antibodies were purchased from Thermo Fisher Scientific. HRP-conjugated secondary antibodies were from Cell Signaling (Goat antirabbit IgG, 1:2000, 7074; mouse antirabbit IgG conformation specific, 1:2000, 3678). Rabbit anti-Human IgG (H + L) HRP-linked Antibody (1:5000, Bethyl, A80-118P).

### Production and Purification of HFn Nanoparticles

Heavy chain apoferritin (HFn) was produced as previously described.^34^ Briefly, the Escherichia coli ClearColi BL21­(DE3) strain (Lucigen Ltd. UK) carrying a pET30b plasmid encoding for HFn was grown at 37 °C in Luria–Bertani medium supplemented with kanamycin until optical density (OD) at 600 nm reached 0.6. Then, the cells were induced with 1 mM of isopropyl β-d-1-thiogalactopyranoside (IPTG). After 3 h, they were collected, washed with phosphate-buffered saline (PBS), and resuspended in a lysis buffer with lysozyme, Benzonase nuclease, and protease inhibitors. After the sonication, the crude extract was heated at 70 °C for 15 min and centrifuged. The supernatant was loaded onto diethylaminoethanol (DEAE) Sepharose anion exchange resin, pre-equilibrated with 2-(*N*-morpholino)­ethanesulfonic acid potassium salt (K-MES) 20 mM, pH 6.0. A stepwise NaCl gradient was used to elute the purified protein. The fractions were analyzed by Sodium Dodecyl Sulfate-PolyAcrylamide Gel Electrophoresis (SDS-PAGE) using 12% (v/v) polyacrylamide gels. The HFn-rich fractions were pulled, loaded on Amicon filters (100 kDa MWCO), and washed several times with PBS to remove traces of small protein contaminants and thereby obtain purer HFn. Protein concentration of the final preparation was determined using the Coomassie Plus Protein Assay Reagent (Thermo Fisher Scientific, MA, USA) and by measuring absorbance at 280 nm. Its purity was assessed by measuring the absorbance ratio 260/280 with a UV–vis spectrophotometer (NanoDrop 2000 Spectrophotometer, Thermo Fisher Scientific). HFn nanoparticles were stored at −80 °C in Tris–HCl 50 mM, pH 7.6, NaCl 150 mM, 10% glycerol to maximize their stability and solubility, as previously described.^35^

### Conjugation and Purification of HFn–CTX Nanoconjugates

The conjugation reaction was adapted from Falvo et al.,^36^ as briefly described below. Amine-containing CTX and sulfhydryl-containing HFn were covalently conjugated by means of a heterobifunctional cross-linker bearing *N*-hydroxysuccinimide (NHS) ester and maleimide (Mal) groups (Malhex–CONH–PEG–NHS, 5 kDa, Rapp Polymere Gmbh, Tubingen). CTX was reacted with a 40-fold molar excess of the cross-linker in phosphate buffer (PBS) pH 7.5 at room temperature for 1 h, and then unreacted species were first removed by washing with PBS buffer in 50 kDa centrifugal filter devices (Amicon, Millipore Corporate) and then on a size-exclusion chromatography (SEC) column (Zeba Spin Desalting Columns, Thermo Fisher Scientific). HFn molecules were added to PEGylated CTX at a final HFn:CTX molar ratio of 1:1 and incubated overnight at 4 °C under stirring. At the end of the reaction, large aggregates were removed by centrifugation (15,000*g* for 15 min at 4 °C). The nanoconjugate-containing supernatant was collected and then purified through two SEC–FPLC steps, the former and the latter employing a Superdex 200 increase 10/300 GL column and a Superose 6 increase 10/300 GL column (GE Healthcare) equilibrated with PBS, respectively, and a flow rate of 0.2 mL min^–1^. The eluted fractions were then characterized with SDS-PAGE, Western blot, and dynamic light scattering (DLS) analyses. The fractions containing the HFn–CTX nanoconjugates were concentrated on Amicon filters (100 kDa MWCO), if deemed necessary. HFn–CTX nanoconjugates were kept at 4 °C in PBS after assessing their stability by means of UV–vis spectrometry and/or centrifugation.

### Labeling of HFn–CTX and HFn Nanoparticles

Double-labeled nanoconjugates were generated by using Alexa Fluor-750-NHS Ester (AF750, Thermo Fisher Scientific) and Alexa Fluor-647-NHS-ester (AF647, Thermo Fisher Scientific).

To label HFn with AF647, a Zeba Spin desalting column (7K MWCO, Thermo Fisher Scientific) was used to exchange the HFn storage buffer for 0.1 M NaHCO_3_ pH 8.0, as per manufacturer’s instructions. AF647-NHS-ester (20 mg mL^–1^ in DMSO) (Thermo Fisher Scientific) was then added to the HFn solution (5 mg mL^–1^) to obtain a molar dye:protein ratio of 50. This mix was stirred at RT for 30 min and then at 4 °C overnight. The unreacted dye was removed with a Zeba Dye and Biotin Removal Columns (7K MWCO, Thermo Fisher Scientific) using 50 mM potassium phosphate, pH 7.2, and 150 mM NaCl as the final buffer. The degree of labeling was calculated as the molar ratio between dye and protein, based on UV–vis spectroscopy. To this end, 280 and 647 nm absorbances were used for the quantification of the protein and the dye, respectively, subtracting the contribution of the dye at 280 nm using the following formula

$$protein\;concentration\;\left(M\right)=\frac{\left[A_{280}-\left(A_{\max}\times 0.03\right)\right]\times df}{\varepsilon_{HFn}\times l}$$

where *A*_max_ is the absorbance (A) of a dye solution measured at the wavelength maximum (λ_max_) for the dye molecule, 0.03 is the correction factor recommended by the supplier to remove the contribution of AF647-NHS-ester to the 280 nm absorbance, df is the dilution factor, which represents the extent (if any) to which the protein:dye sample was diluted for the absorbance measurement, l (optical path length) is 0.1 cm, and ε_HFn_ is 458,340 M^–1^ cm^–1^ (calculated with ProtParam).^37^

$$Degree\;of\;labeling\;\left(DOL\right)=\frac{moles\;Alexa\;647}{moles\;HFn}=\frac{A_{\max}\times df}{\varepsilon_{Alexa\;647}\times 1\times M}$$

where ε_Alexa 647_ (molar extinction coefficient of AF-647-NHS-ester) is 239,000 M^–1^ cm^–1^ and *M* is the protein concentration. HFn-647 was stored at −80 °C in a previously described buffer.^38^

CTX was labeled with AF750 using the same procedure and similar reaction conditions. In this case, AF750-NHS-ester (10 mg mL^–1^ in DMSO) (Thermo Fisher Scientific) was added to the CTX solution (5 mg mL^–1^) to obtain a molar dye:protein ratio of 10. Before starting the reaction, a 40-fold molar excess of the PEG cross-linker was added to the reaction mix because both AF750 and PEG cross-linker compete to bind to the amine groups of CTX. Then the reaction was kept under stirring for 2.5 h at RT. At the end of the incubation, the unreacted dye was removed and labeled CTX was further purified using SEC–FPLC as described above. Quantification of the protein and the dye was performed using the formula reported above (0.04 is the correction factor recommended by the supplier to remove the contribution of the AF750-NHS-ester from the 280 nm absorbance, ε_Alexa 750_ is 240,000 and ε_CTX_ is 217,440 M^–1^ cm^–1^ as calculated by ProtParam).

### Cell Culture

U-87 MG (ATCC, HTB-14) was cultured in high glucose Dulbecco’s Modified Eagle’s Medium (DMEM) supplemented with 10% fetal bovine serum (FBS), 2 mM l-glutamine, penicillin (50 IU mL^–1^), and streptomycin (50 mg mL^–1^). MDA-MB-231 cells (Amsbio, SC059-Puro) were cultured in high glucose DMEM supplemented with 10% fetal bovine serum (FBS), 2 mM l-glutamine, penicillin (50 IU mL^–1^), streptomycin (50 mg mL^–1^), and 0.1 mM MEM with nonessential amino acids (NEAA).

All cell lines were maintained at 37 °C in a humidified atmosphere containing 5% CO_2_ and passaged prior to confluence. Cells were regularly tested for mycoplasma, and all experiments were performed on mycoplasma-free cells.

### Spheroids Generation

U-87 MG cells were detached with trypsin–EDTA and the cell number was determined using a Bürker chamber. The cell suspension was diluted to 6 × 10^4^ cells mL^–1^ in complete medium. A volume of 100 μL of the cell suspension (6000 cells) was added into each well of an ultralow attachment Nunclon Sphera 96-well plate (Thermo Fisher Scientific). Spheroid formation was initiated by spinning the plates at 340*g* for 10 min using an Eppendorf 5810 centrifuge (Eppendorf AG, Hamburg) with swinging buckets. The plates were incubated at 37 °C and 5% CO_2_ in a humidified incubator for 5 days.

MDA-MB-231 cells were detached and counted as described above. The cell suspension was diluted to 2.5 × 10^4^ cells mL^–1^ in ice-cold medium. Cultrex (3-D Culture Matrix Reduced Growth Factor BME, R&D SYSTEM) was thawed on ice overnight and added with ice-cold pipet tips at a final concentration of 2.5% to the cell suspension. A volume of 200 μL of the cell suspension (5000 cells) was added into each well of an ultralow attachment Nunclon Sphera 96-well plate (Thermo Fisher Scientific). Spheroid formation was initiated as above, and the plates were incubated at 37 °C and 5% CO_2_ in a humidified incubator for 5 days. Spheroid area was measured at the Incucyte (Sartorius) by using the built-in spheroid analysis module.

### Isolation of Effector Cells

Peripheral blood mononuclear cells (PBMCs) were obtained by centrifugation on Ficoll–Paque of blood samples (50 mL) from healthy human donors (408*g* without breaks for 30 min at RT). The PBMCs layer was carefully transferred into a new 50 mL tube, diluted with PBS, and centrifuged at 355 for 6 min at RT. Then, the supernatant was discarded to remove platelets, and this procedure was repeated four times decreasing the centrifugation speed down to 180*g*. Washed PBMCs were resuspended in RPMI-1640 medium supplemented with 10% of heat-inactivated FBS and 1,000 U mL^–1^ IL-2 (BioLegend, San Diego, CA, USA) for 24 h at 37 °C. This procedure was approved by the Ethical Committee of the University of Milano-Bicocca after the submission of the project together with informed consent by the healthy volunteers (prot. 0138485/21, November 15th, 2021).

### Caspase Activation Assay

For end point assays, apoptosis of target cells was evaluated using the Caspase-Glo 3/7 3D Assay (Promega Corporation, Madison, WI, USA). Spheroids were first washed and transferred in FBS-free RPMI culture medium, prior to treatment with free CTX, or HFn–CTX nanoconjugates (10 μg mL^–1^), or HFn (34 μg mL^–1^), all diluted in FBS-free RPMI culture medium to reach a final volume of 0.2 mL. Note that HFn was given at 34 μg mL^–1^, as 10 μg mL^–1^ of CTX in the nanoconjugate formulation corresponds to 34 μg mL^–1^ of HFn due to the 1:1 molar ratio between the two protein species. After incubation at 4 °C for 30 min, IL-2-activated PBMCs were added onto the target cells at an effector:target (E:T) ratio of 2:1 and incubated at 37 °C for 24 or 48 h. Then, 100 μL of Caspase-Glo 3/7 3D Reagent was added to each well, and the plate was incubated at RT for at least 30 min. Luminescence, which is proportional to the amount of caspase activity, was measured with an EnSight multimode plate reader (PerkinElmer, Waltham, MA, USA).

### HFn–CTX and HFn Uptake Assays

U-87 MG and MDA-MB-231 cells were seeded onto 4-compartment 35 mm glass bottom Petri dishes (Greiner Bio-One) at a density of 130,000 cells and 60,000 cells per compartment, respectively, in 0.5 mL of complete medium. On the following day, the medium was replaced with 0.2 mL of fresh complete medium containing HFn–AF647–CTX–AF750 (double-labeled nanoparticles) or HFn–AF647 (single-labeled nanoparticles). The cells were then incubated for 0, 2, 15, 30, 60, and 120 min at 37 °C.

For the 2D cultures, the growth medium was removed, and the cells were washed twice with prewarmed PBS. Subsequently, they were fixed and immunostained as previously described.^39,40^ Nuclei were stained with Hoechst (5 μg mL^–1^) for 5 min at RT. Cells were then rinsed once with PBS before being imaged using a fluorescence microscope (Thunder Imager Live Cells, LEICA Microsystem).

For the 3D cultures, spheroids were treated with HFn–AF647–CTX–AF750 or HFn–AF647 for 30 min, 1 h, and 4 h before cutting them into slices. As a control, a parallel set of spheroids was treated with equal moles of AF750-labeled CTX for 1 h. For the spheroid cryosections, Dakopen was used to draw a circle around the sectioned spheroids to create a water-repellent barrier. Glass slides were washed in PBS prior to adding the blocking and permeabilization buffer (0.2% BSA + 0.1% Triton X-100 in PBS) at RT for 10 min. After three washes with PBS, immunostaining was performed by incubating slices with primary antibodies diluted in PBS, 1% BSA at RT for 1 h. Next, spheroid slices were rinsed thrice with PBS prior to the addition of secondary antibodies diluted in PBS, 1% BSA at RT for 45 min in the dark. After three washes with PBS, nuclei were stained with Hoechst (5 μg mL^–1^) by adding 50 μL on each drawn circle and incubating at RT for 5 min. Spheroid sections were then washed three times with PBS and covered with a coverslip on which a drop of FluorSave Reagent (Millipore Corporation) was deposited. Mounted cryosections were dried at 37 °C for 20 min and then imaged using a fluorescence microscope (Thunder Imager Live Cells, LEICA Microsystem). Identical settings were used to obtain different experimental groups.

### Embedding and Cryosectioning of Spheroids

Spheroids were fixed in prewarmed 4% paraformaldehyde in PEM buffer (80 mM PIPES pH 6.8, 5 mM EGTA, 2 mM MgCl_2_)^35,40^ for 2 h at RT (200 μL in each of the 96 wells), washed once with PBS w/o Ca^2+^ and Mg^2+^, and then dehydrated with 30% sucrose for 2 h at RT. At the end of the incubation, the spheroids were cryopreserved in OCT compound (R0030, Histo-Line laboratories) exploiting a freezing bath filled with ethanol and dry ice.^41^ Frozen samples were then stored at −80 °C until sectioning. Before being sectioned, embedded spheroids were left at −20 °C for at least 1 h. Slices of 7 μm were cut using an MC4000 cryostat (Histo-Line laboratories) and collected on SuperFrost Plus glass slides (HL26766, Histo-Line laboratories), which were stored at −80 °C for further use.

### Cell-Surface EGFR and TfR1 Expression Levels

U-87 MG and MDA-MB-231 cells were co-seeded onto 35 mm glass-bottom Petri dishes (Greiner Bio-One), each at a density of 70,000 cells, in 1.25 mL of complete medium. Before plating, MDA-MB-231 cells were stained with the Vybrant CFDA SE Cell Tracer Kit (Thermo Fisher Scientific) according to the manufacturer’s instructions. On the following day, cocultures were fixed and immunostained using either anti-EGFR or anti-TfR1 antibodies. To stain for total or only cell-surface receptors, cells were blocked with or without Triton X-100 as a permeabilizing agent, respectively. Nuclei were stained with Hoechst (5 μg mL^–1^) for 5 min at RT. Non-permeabilized samples stained for a cytosolic marker, typically actin, provided a control for the integrity of the specimens and for the specific detection of cell-surface signals (not shown). Cells were then rinsed once with PBS prior to imaging at a Thunder Imager Live Cells microscope (LEICA Microsystem), using identical acquisition settings. The images were then analyzed with CellProfiler to quantify cell-surface EGFR and TfR1 levels.

### Image Processing and Analysis

Colocalization analysis was performed using Manders’ colocalization plugin of ImageJ (National Institutes of Health, Bethesda, MD, USA). This coefficient measures the pixel-by-pixel covariance in the signal levels of two images.^42^ The background was subtracted before analysis using a rolling ball of 15.0 pixels. The colocalization analysis was performed on three sections for which Manders’ coefficients were calculated with automatic threshold, which is algorithmically determined by optimizing the separation between signal and noise. This approach reduces the influence of artifacts and nonspecific signal intensities, providing a more realistic and robust representation of molecular colocalization (tM = 0 means no colocalization, tM = 1 means completed colocalization). For display, images of the cryosections were contrasted in Adobe Photoshop, using identical settings.

CellProfiler was used to write bespoke pipelines for the automated analysis of spheroids’ cryosections and cell-surface receptor levels.^43^

### Western Blot

To quantify TfR1 and EGFR expression, MDA-MB-231 and U-87 MG cells were washed three times with ice-cold PBS, detached from Petri dishes with Trypsin (0.05%), and collected by centrifugation. They were then lysed with RIPA buffer supplemented with a Halt Protease Inhibitor Cocktail (Thermo Fisher Scientific). The lysates were incubated at 4 °C for 30 min on a rocking wheel and then centrifuged at 13,000*g* at 4 °C for 15 min. Proteins in the cleared lysates were quantified by using the bicinchoninic acid assay (QPRO-BCA Kit Standard, Cyanagen). 15 μg of total proteins was separated by SDSPAGE, and then transferred onto a PVDF membrane (0.45 μm pore size; Whatman, GE Healthcare) as previously described.^38,44^ Membranes were blocked in TBS supplemented with Tween 20 0.1% and BSA 5% for 1 h at RT and incubated with primary antibodies according to the manufacturer’s instructions, followed by HRP-conjugated secondary antibodies. The signal was acquired with a ChemiDoc Imaging System (Bio-Rad, USA).

### Densitometry

To estimate band intensities, nonsaturated exposures of Western blots were subjected to densitometric analyses using ImageJ. Normalized TfR1 and EGFR expression was determined as follows: the intensities of TfR1 or EGFR and those of a housekeeping protein (e.g., actin) were determined by densitometry, and the TfR1 or EGFR/actin ratio was calculated as previously described.^35^

### Tumor Xenografts

Female NOD/SCID mice purchased from Envigo (Bresso, Italy) were housed in a fully equipped facility according to the guidelines of the Italian Ministry of Health. Experiments were conducted under an approved protocol (authorization n° 92/2022-PR). Luciferase-expressing MDA-MB-231 cells were cultured in high glucose DMEM supplemented with 10% fetal bovine serum, 2 mM l-glutamine, penicillin (50 IU mL^–1^), streptomycin (50 mg mL^–1^), and 0.1 mM MEM with nonessential amino acids (NEAA). A 1:1 mixture comprising 2 × 10^6^ cells and Matrigel (Corning Matrigel Growth Factor Reduced (GFR) Basement Membrane Matrix, Phenol Red-free, LDEV-free) was inoculated subcutaneously into the mouse mammary fat pad in a final volume of 100 μL. The health and behavior of these mice were observed daily, and the weight was monitored every 3 days until the tumors reached a volume around 100 mm^3^ (typically 3 weeks after inoculation) as measured with a caliper.

The tumors’ dimension was also evaluated by means of the bioluminescence (BLI) signal in vivo, which was measured using an IVIS Lumina II imaging system (PerkinElmer), with an exposure time of 1 min. Twenty-four hours before injecting the nanoparticles, BLI images were acquired 5, 8, and 10 min after peritoneal injection of 260 μg kg^–1^ luciferin (d-luciferin potassium salt, PerkinElmer). Bioluminescence measurements were used to divide mice into experimental groups with similar tumor burden.

### Tumor Targeting and Biodistribution of HFn–CTX In Vivo and Ex Vivo

To study the biodistribution of HFn–CTX in mice, the nanoconjugate was labeled with AF647. NOD/SCID mice bearing luciferase-expressing MDA-MB-231 cell-derived tumors were immobilized in a restrainer (2Biological Instruments). Next, HFn–AF647–CTX (5 mg kg^–1^ body weight) was injected into the tail vein with a 26G insulin needle, with PBS being used as a control. Another group of mice was injected with equal moles of free AF750-labeled CTX. Epifluorescence images of anesthetized mice were obtained 30 min, 1 h, 3 h, and 24 h post injection at the IVIS system (PerkinElmer), keeping the animals at 37 °C. Images were acquired with a Cy5.5 emission filter, while excitation was scanned from 570 to 640 nm, and autofluorescence was removed by spectral unmixing. The instrument setup for FLI acquisition is Fstop 2 and medium binning, exposure time of 30 s. After being imaged, those mice were sacrificed, and organs were dissected and imaged with the IVIS system at RT. All epifluorescence (Epf) intensity values were normalized after subtracting Epf values obtained from mice injected with PBS, and the results were expressed as the mean ± standard errors (s.e.).

Among off-target organs, the liver, lungs, and kidneys exhibited higher accumulation of the nanoconjugate as determined by IVIS analysis. Tissue samples at a concentration of 250 mg mL^–1^ in PBS were mechanically homogenized using a TissueLyser II (QIAGEN) at a frequency of 30 s^–1^ and beads for 15 min at 4 °C. Fluorescence at 665 nm was measured using an EnSight multimode plate reader (PerkinElmer, Waltham, MA, USA) and from 100 μL of each suspension. This signal, upon subtraction of the blank (i.e., corresponding control mouse tissue), was correlated with the concentration of nanoconjugate by spiking the corresponding control mouse tissues with known concentrations of HFn–AF647–CTX. By measuring the weight of each organ and knowing the injected dose of nanoconjugate, we calculated the percentage of injected nanoconjugate present at different time points (ID %, Injected Dose Percentage).

Tumors were processed using the same method at a concentration of 200 mg mL^–1^ with RIPA buffer for lysis.

Plasma from each mouse was obtained by spinning at 2,980*g* for 15 min at 4 °C blood samples collected prior to sacrifice in heparinized tubes. Fluorescence at 665 nm was measured and correlated with the concentration of the nanoconjugate as described above.

### Statistical Analysis

Statistical analyses were conducted using adequate tests, including *t*-test, one-way ANOVA, and two-way ANOVA. Graphs show mean values ± standard deviation (s.d.) or standard errors (s.e.). All tests assumed normal distribution and the statistical significance threshold was set at *p* < 0.05.

## Results

### Production and Biophysical Characterization of CTX-Conjugated HFn Nanoparticles

To produce the HFn–CTX nanoconjugate, we initially employed a standard protocol for mAbs-conjugated HFn nanoparticles.^24,36^ In brief, HFn nanoparticles were produced and purified using established methods,^34^ whereas CTX was reacted with a 5 kDa PEG-based heterobifunctional cross-linker harboring one *N*-hydroxysuccinimidyl ester (NHS) and one maleimide (Mal) group. Next, the resulting PEGylated antibody was conjugated with HFn nanoparticles to synthesize the HFn–CTX nanoconjugate (Figure 1A). We optimized the stoichiometries of the various reactants, exploiting those used for the production of other HFn–mAbs nanoconjugates as a reference point.^24^ For the HFn–CTX nanoparticles, a 1:1 molar ratio between the two protein species was selected to ensure that both HFn and CTX would be adequately spaced to bind their cognate receptors and to minimize accumulation in the liver.^45^ Utilizing these conditions, batches of HFn–CTX nanoparticles with reproducible physicochemical properties could be synthesized (Table S1).

**Figure 1:**
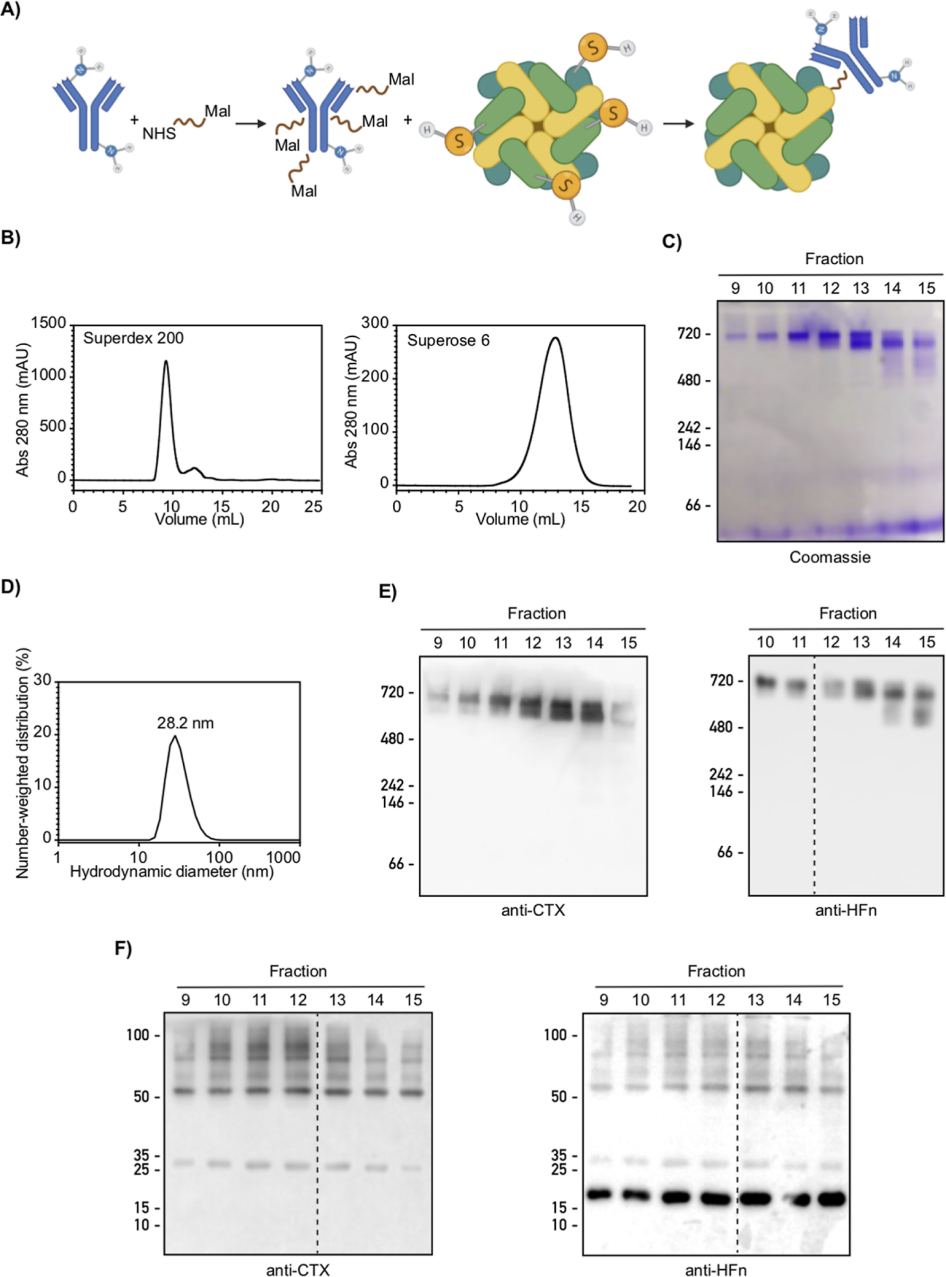
Synthesis, purification, and characterization of the HFn–CTX nanoconjugate. (A) Schematic representation of the two reactions leading to the synthesis of the HFn–CTX nanoconjugate. PEGylation of the mAb is followed by conjugation with HFn nanoparticles. (B) Representative elution profiles of the purification of the HFn–CTX nanoconjugate by SEC-FPLC. (Left) Elution profile of the products obtained from the synthetic reaction (A) on Superdex 200. (Right) Elution of the void fractions (9–11) from the Superdex 200 column loaded on a Superose 6. (C,E) Characterization of the native HFn–CTX nanoconjugate. Protein molecular weight markers, purified HFn and CTX standards, and equal volumes (10 μL) from the indicated fractions (fr) of the HFn–CTX complex eluted from the Superose 6 column were separated by native page electrophoresis (running gel: 6%) and blotted with the indicated antibodies. (D) Size of the HFn–CTX nanoconjugate. DLS was used to determine the hydrodynamic size of the purified HFn–CTX nanoconjugate. (F) Characterization of the denatured HFn–CTX nanoconjugate. The fractions analyzed in (C) were separated by SDS-PAGE (running gel: 10%) and blotted as indicated. Dashed lines indicate removal of intervening lanes.

We compared different HFn vs Alexa Fluor 488 (AF488) dye molar ratios, as well as different protein concentrations and reaction conditions, to optimize protein labeling (Table S2). Based on the degree of labeling (DOL), we chose a 1:50 molar ratio and an HFn concentration of 5 mg mL^–1^. Similar experiments were conducted to optimize the labeling of CTX, using AF647 dye (Table S3). PEG was added to the reaction because it competes with AF647 for the binding to the amine groups of CTX. We selected a 1:10 molar ratio and a CTX concentration of 5 mg mL^–1^ (Table S3) to avoid overlabeling, which could enhance aggregation while reducing antibody specificity. This streamlined the production of different variants of fluorescent nanoconjugates, among which we characterized in vitro and in vivo those labeled on HFn and CTX with AF647 and AF750, respectively, for their advantageous emission spectra (Tables S4 and S5).

The nanoparticles were purified by size exclusion chromatography (SEC) and subsequently analyzed by SDS-PAGE and Western blot (Figure 1B–E). We initially took advantage of the SEC setup utilized for HFn–trastuzumab nanoconjugates,^24^ but we were unable to identify distinct peaks or fractions rich in the HFn–CTX nanoconjugate while depleted of each unreacted species (Figure S1A). Hence, we established a new two-step purification protocol, described in Figure S1B, to retrieve good amounts of highly pure HFn-CTX nanoconjugates (Figure 1C,E,F). The hydrodynamic size of this nanoconjugate was lower than 30 nm, as measured by dynamic light scattering (DLS) (Figure 1D).

### HFn–CTX Nanoconjugate Boosts ADCC in TNBC Spheroids

To evaluate ADCC induced by the HFn–CTX nanoconjugate, we quantified apoptosis induced by human peripheral blood mononuclear cells (PBMCs), which contain NK cells. Upon activation, NK cells polarize and exocytose Perforin and Granzyme B granules, which induce apoptotic cell death by causing the cleavage of procaspase 8/10 and the ensuing activation of caspase 3/7.^46^

GBM and TNBC spheroids consisting of U-87 MG or MDA-MB-231 cells, respectively, were preincubated with either CTX, or HFn–CTX, or HFn, prior to adding IL-2-activated PBMCs. The spheroids were lysed 24 and 48 h later, and caspase 3/7 activity was measured. The TNBC spheroids treated with the nanoconjugate showed a more prominent caspase activation than those treated with either CTX or HFn alone (Figure 2A, right). Unexpectedly, ADCC was much milder in the GBM spheroids, and only the CTX-treated ones exhibited a weak yet significant response (Figure 2A, left). Importantly, the ADCC enhancement observed in the TNBC spheroids was independent of spheroid size (Figure 2B) or HFn–CTX penetration, as shown by automated image analysis of cryosections obtained from parallel GBM and TNBC spheroids (Figure S2). This aligns with the observation that NKs do not heavily infiltrate the core of TNBC tumors.^47^ The HFn–CTX nanoconjugate was mainly detected in the outer layers of the spheroids, whereas the HFn nanoparticles were slightly more infiltrated, particularly in the MDA-MB-231 spheroids (Figure S2A,B). The detected signals were specific, as shown by imaging untreated spheroids (Figure S2C). Of note, accumulation and distribution within spheroids did not seemingly change by increasing the incubation time from 30 min to 4 h (Figure S2A,B) and were similar to that of free CTX (Figure S3A). These results are consistent with the receptor-mediated uptake of both HFn and CTX and with CTX conjugation further enhancing high-affinity binding of HFn nanoparticles on the cell surface.

**Figure 2:**
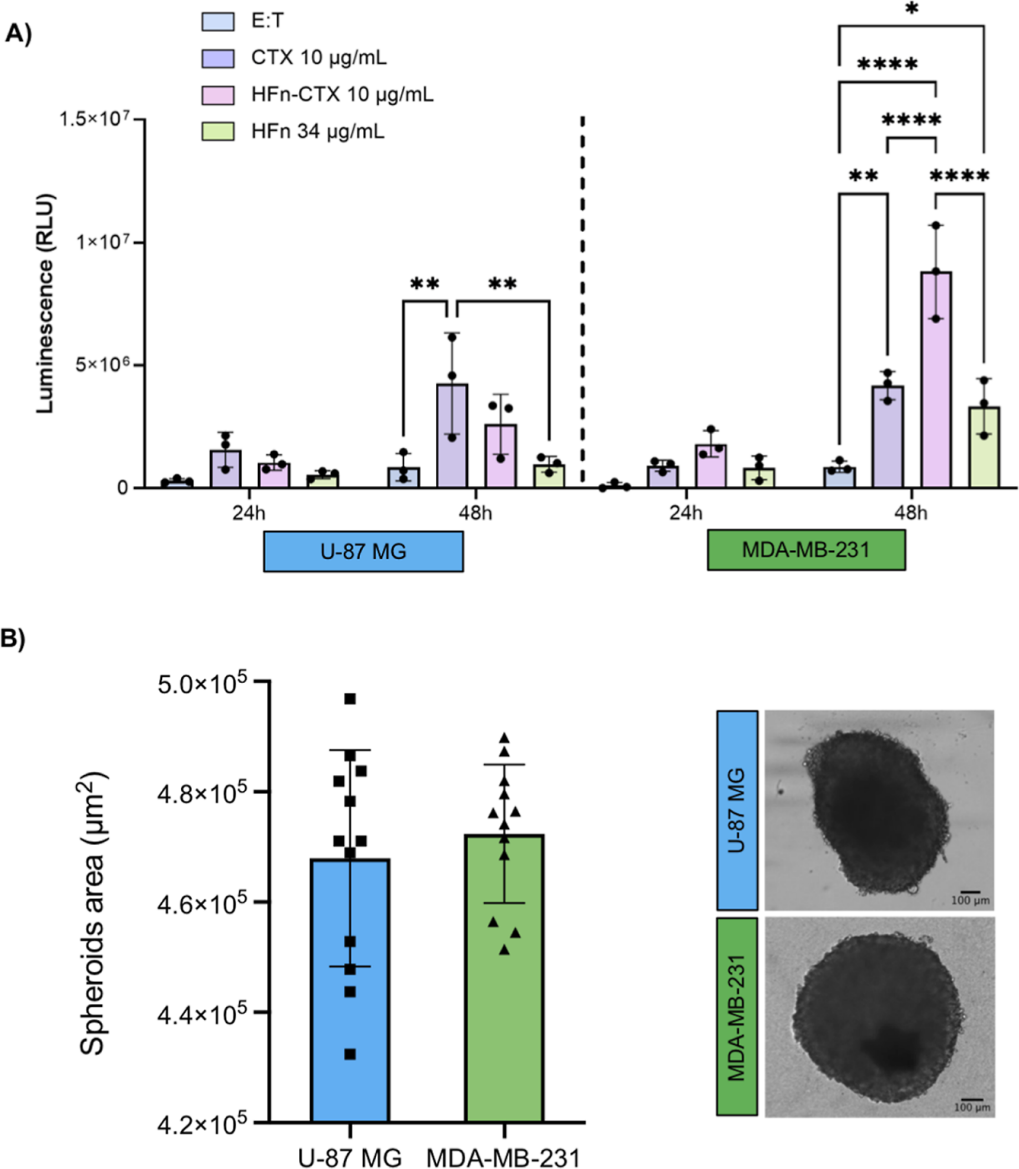
The HFn–CTX nanoconjugate promotes ADCC in TNBC but not GBM spheroids. (A) Caspase activity in U-87 MG and MDA-MB-231 spheroids treated with CTX (10 μg mL^–1^), or HFn–CTX (10 μg mL^–1^), or HFn (34 μg mL^–1^) was measured at 24 and 48 h upon addition of IL-2-activated PBMCs. Bar graphs show the mean ± s.e. of 3 independent replicates (each composed of three technical replicates). * = *p* < 0.05; ** = *p* < 0.01; **** = *p* < 0.0001 (two-way ANOVA). (B) Area of U-87 MG and MDA-MB-231 spheroids. Each square or triangle represents the area of a single spheroid at 5 days of seeding. On the right, representative images of U-87 MG and MDA-MB-231 spheroids at 5 days of seeding.

### Uptake of the HFn–CTX Nanoconjugate Is Slower in TNBC than in GBM Cells

To gain insight into why the HFn–CTX nanoconjugate promoted robust ADCC in TNBC but not in GBM spheroids, we analyzed its uptake and compared it to that of the HFn nanoparticles. The TfR1 is known to undergo rapid and constitutive internalization via clathrin-mediated endocytosis and then recycles back to the plasma membrane,^48,49^ whereas the EGFR can be internalized through different routes, which contribute to determining whether it is recycled or degraded.^50,51^

Time-course experiments revealed that the HFn–CTX nanoconjugate was rapidly internalized in U-87 MG cells, where it localized in punctate structures likely corresponding to the endolysosomal system, as early as at 5 min of incubation (Figures 3A and S4). Interestingly, the nanoconjugate and HFn nanoparticles showed similar uptake kinetics (Figure 3A). Surprisingly, the nanoconjugate lingered on the plasma membrane of MDA-MB-231 cells much longer (up to 30 min), and it took longer to accumulate in intracellular compartments compared to the HFn nanoparticles (Figures 4A and S4).

**Figure 3:**
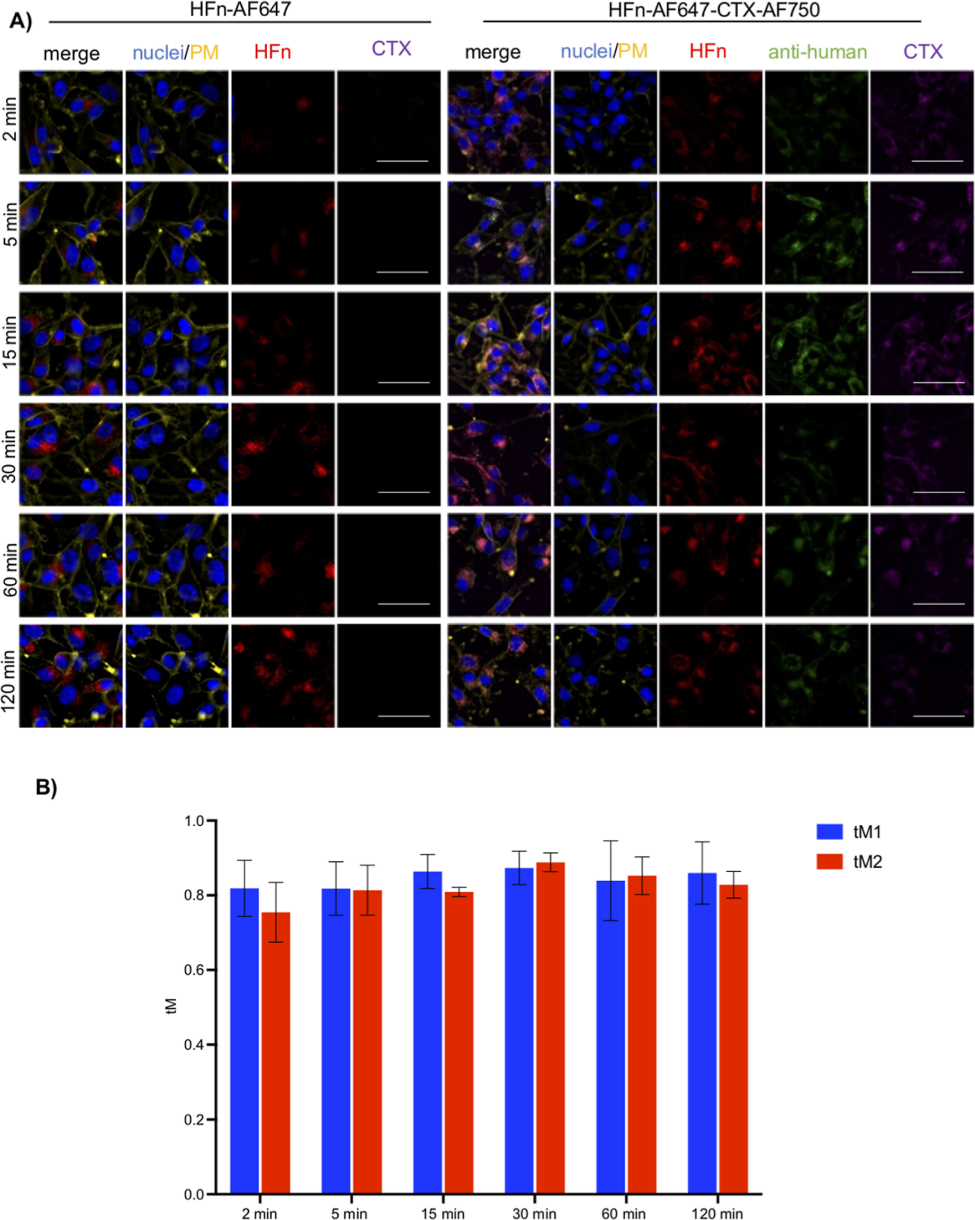
The HFn–CTX nanoconjugate and the HFn nanoparticles show similar uptake kinetics in GBM cells. (A) Representative images of U-87 MG cells incubated with double-labeled HFn–CTX or labeled HFn (0.1 mg mL^–1^ each) for 2, 5, 25, 30, 60, and 120 min. HFn–AF647 is depicted in red, CTX in magenta, and CTX detected with anti-human-AF488 in green. The plasma membrane (PM) was stained with CD44 (yellow), and nuclei were stained with Hoechst (blue). Scale bar, 100 μm. (B) Manders’ coefficient analysis on the HFn–CTX nanoconjugate and HFn nanoparticles. The bar graph shows tM1 (CTX-AF488 signal vs HFn–AF647 signal, blue bars) and tM2 (HFn–AF647 signal vs CTX-AF488 signal, red bars) at the indicated time points.

**Figure 4:**
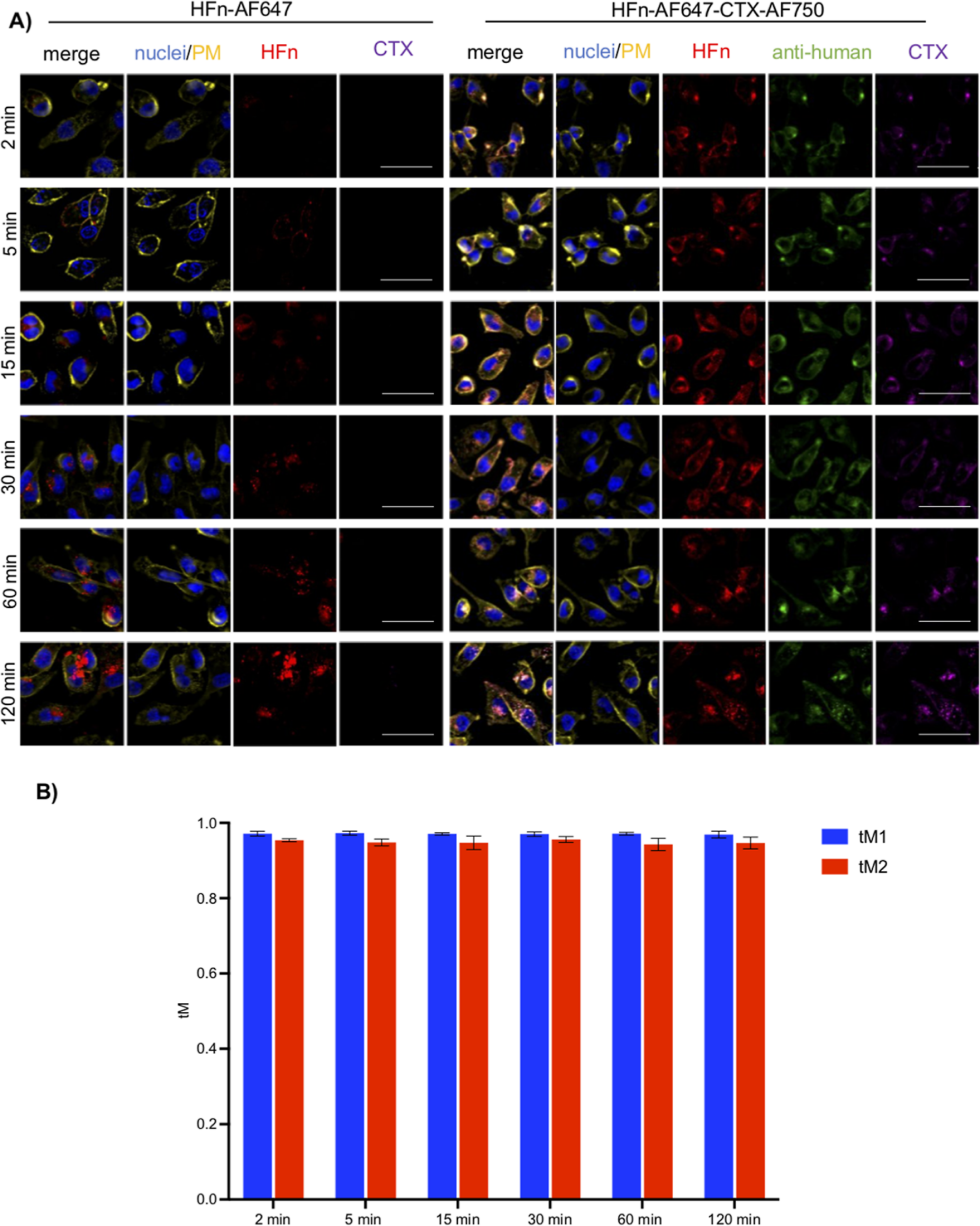
TNBC cells take up the HFn–CTX nanoconjugate more slowly than the HFn nanoparticles. (A) Representative images of MDA-MB-231 cells incubated with double-labeled HFn–CTX or labeled HFn (0.1 mg mL^–1^ each) for 2, 5, 25, 30, 60, and 120 min. Cells were processed, stained, and imaged as in Figure 3A. Scale bar, 100 μm. (B) Manders’ coefficient analysis at all the tested time points. Data were obtained and plotted as in Figure 3B.

Manders’ coefficient values (tM1 = CTX-AF488 signal vs HFn-AF647 signal and tM2 = HFn-AF647 signal vs CTX-AF488 signal) were close to 1 at all the tested time points in both cell lines, indicating an almost complete colocalization between the HFn and CTX signals (Figures 3B and 4B). These results ruled out that a cell type-specific massive dismantling of the internalized nanoconjugate could explain the above discrepancies.

Hence, the MDA-MB-231 cells seem to internalize the HFn–CTX nanoconjugate more slowly than the HFn nanoparticles, whereas both are taken up faster and with similar kinetics by the U-87 MG cells.

### TNBC and GBM Cells Have Distinct Cell-Surface Levels of EGFR and TfR1

Differences in the expression or cell-surface levels of the receptors to which HFn and CTX bind, TfR1 and EGFR, respectively, would impact the internalization of the nanoconjugate and thereby account for the above observations. Thus, we sought to compare the protein levels of TfR1 and EGFR in glioblastoma and TNBC cells. Western Blot analyses showed that TfR1 was significantly more expressed in the U-87 MG than in the MDA-MB-231 cells, whereas the opposite held for the EGFR (Figure 5A,B). Furthermore, measuring the fluorescence intensity of the HFn–CTX nanoconjugate or HFn nanoparticles at early time points (2 min) of uptake, when the degradation of internalized cargos has not yet occurred, unmasked that the signal of the bound HFn–CTX nanoconjugate was not only much higher (74×) but also increased (3×) compared to that of the HFn nanoparticles bound to the MDA-MB-231 or U-87 MG cells (Figure 5C). These data suggest that the former cells have more binding sites for these nanoparticles.

**Figure 5:**
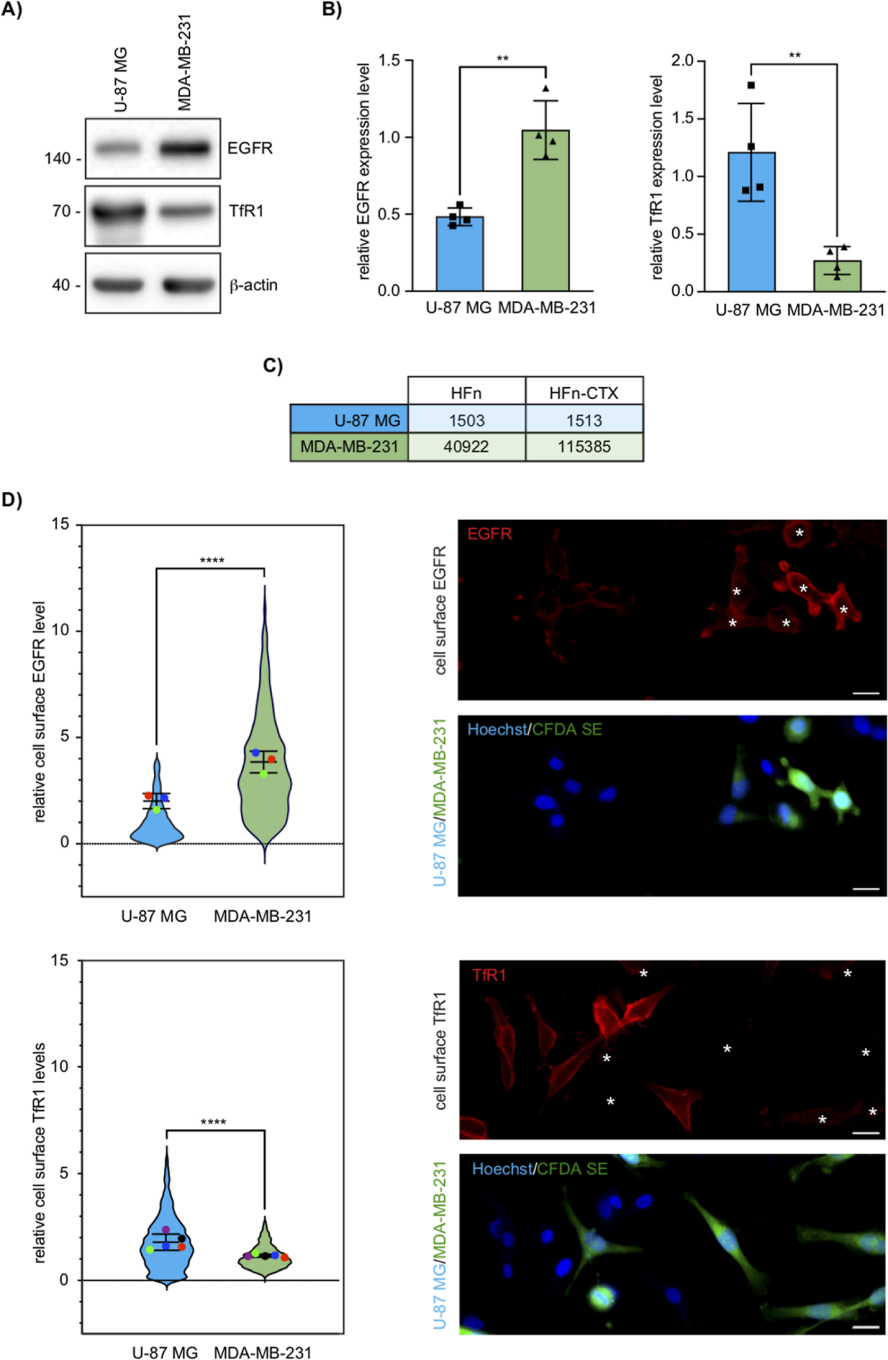
TfR1 and EGFR expression in U-87 MG and MDA-MB-231 cells. (A) Representative Western blot of total lysates of U-87 MG and MDA-MB-231 cells (15 μg) blotted as indicated. (B) Densitometric quantification of the TfR1 and EGFR signal normalized on the corresponding β-actin signal. Bar graphs show mean ± s.e. of 3 independent replicates. ** = *p* < 0.01 (two-tailed Student’s *t*-test). (C) Quantification of the fluorescence intensity of HFn alone or conjugated with CTX normalized by the number of nuclei. Data were obtained by measuring HFn–AF647 fluorescence in U-87 MG and MDA-MB-231 cells after 2 min of incubation with HFn or HFn–CTX. (D) Cell-surface EGFR and TfR1 levels as measured with CellProfiler and representative images of non-permeabilized cells stained for EGFR or TfR1. Nuclei were stained with Hoechst (blue), TfR1 and EGFR (red), and CFDA SE-labeled MDA-MB-231 cells (green). White asterisks mark the position of the MDA-MB-231 cells in the anti-TfR1 and anti-EGFR micrographs. Scale bar, 20 μm. Violin superplots show the mean ± s.e. of 3 independent replicates (1665 U-87 MG cells and 1065 MDA-MB-231 cells for EGFR; 2178 U-87 MG cells and 1751 MDA-MB-231 cells for TfR1). ** = *p* < 0.01; ****p < 0.0001 (unpaired two-tailed *t*-test).

To follow up on this, we measured the relative amount of cell-surface EGFR and TfR1 in MDA-MB-231 and U-87 MG co-cultures by staining non-permeabilized fixed specimens with antibodies targeting the ectodomain of either receptor. Automated quantitative analysis of the resulting microscopy images revealed that MDA-MB-231 cells had more EGFR on the cell surface than U-87 MG cells. Conversely, cell-surface TfR1 showed the opposite trend, although differences were slightly less marked (Figures 5D and S5).

### Biodistribution of the HFn–CTX Nanoconjugate and Tumor Targeting

Prompted by the sum of the above results, we sought to assess the biodistribution of the HFn–CTX nanoconjugate and its ability to target TNBC tumors. MDA-MB-231 cells stably expressing a luciferase reporter were injected subcutaneously into female NOD/SCID mice. When tumors reached a volume of 100 mm^3^, typically 3 weeks post-injection, the AF647-labeled HFn–CTX nanoconjugate (5 mg kg^–1^) was injected into the tail vein, whereas PBS was used as a control. To evaluate biodistribution, healthy tumor-bearing mice were administered the HFn–CTX nanoconjugate, and epifluorescence (Epf) images were taken at 30 min, 1 h, 3 h, and 24 h posti-njection, keeping the animals at 37 °C. Next, the mice were sacrificed; organs and tumors were dissected and subsequently imaged at room temperature. The signal of the HFn–CTX nanoconjugate was intense in the liver, in line with the known biodistribution of HFn nanoparticles.^52^ For this reason, it was masked, while whole animals were imaged (Figure 6A). Resected tumors showed a progressive accumulation of the nanoconjugate from 30 min to 3 h, when the signal peaked, followed by a marked decrease at 24 h post-injection (Figure 6B). The fluorescence measured in excised organs confirmed that a prevalent fraction of the HFn–CTX nanoconjugate not trapped in the tumor mass was rapidly sequestered by the liver, as previously described.^52,53^ Notably, the liver exhibited a detectable emission at all time points (Figure 6C). Compared to the nanoconjugate, free CTX showed a higher accumulation in tumors but not in the liver (Figure S3B). Besides the liver, the lungs and the spleen showed the presence of the HFn–CTX nanoconjugate until 3 h post-injection (Figure 6C).

**Figure 6:**
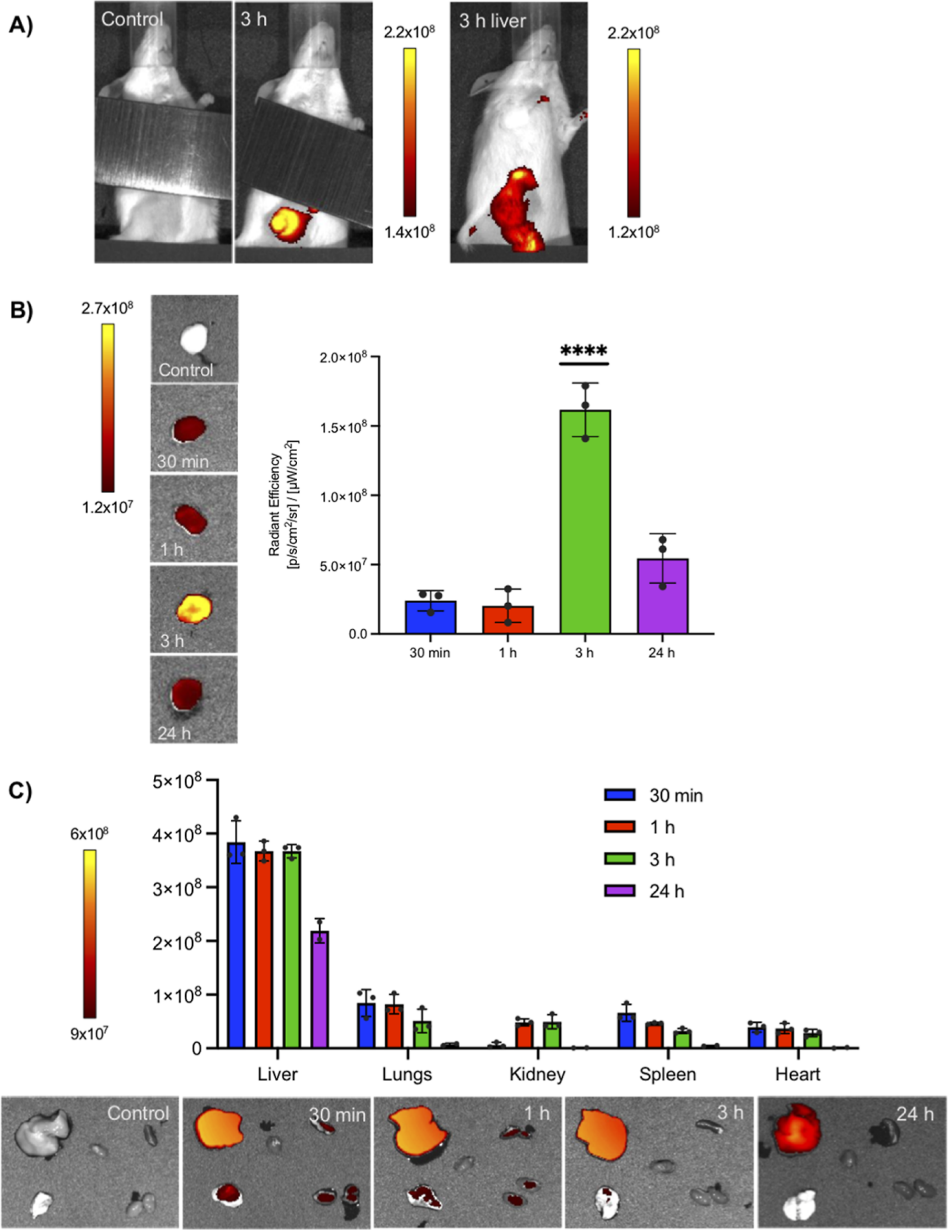
The HFn–CTX nanoconjugate displays efficient long-lasting tumor targeting. (A) (Left) Representative epifluorescence (Epf) images of tumor-bearing mice taken 3 h (3 h) after intravenous injection of 5 mg kg^–1^ HFn–AF647-CTX or buffer alone (control). A ruler (visible as a gray bar in the pictures) was put on the belly to mask the signal deriving from the accumulation of the nanoconjugate within the liver. (Right) Representative Epf images of tumor-bearing mice taken 3 h after intravenous injection showing also the signal derived from the liver. (B) Epf of isolated MDA-MB-231 tumors and averaged Epf intensity of tumor region of interest (ROI) acquired 30 min, 1 h, 3 h, and 24 h after HFn–CTX injection. (C) Epf of isolated spleen (S), kidneys (K), liver (L), heart (H), lungs (Lu), and averaged Epf intensity of the ROI obtained after 30 min, 1 h, 3 h, and 24 h after HFn–CTX injection. The color scale in (A–C) indicates the averaged epifluorescence expressed as radiant efficiency [(p/s/cm^2^/sr)/(mW/cm^2^)], where p/s/cm^2^/sr is the number of photons per second that leave a cm^2^ of tissue and radiate into a solid angle of one steradian (sr). Values reported in (B,C) are the mean ± s.e. of 3 independent samples for each experimental condition. **** = *p* < 0.0001 (one-way ANOVA).

Confocal imaging of tumor cryosections corroborated that the HFn–CTX nanoconjugate started to accumulate in the tumor mass early after injection and was no longer visible at 24 h (Figure 7A). Given the size of the nanoconjugate and CTX (∼30 vs 15 nm), it is not surprising that free CTX appeared to diffuse farther from perivascular regions (Figure 7B).

**Figure 7:**
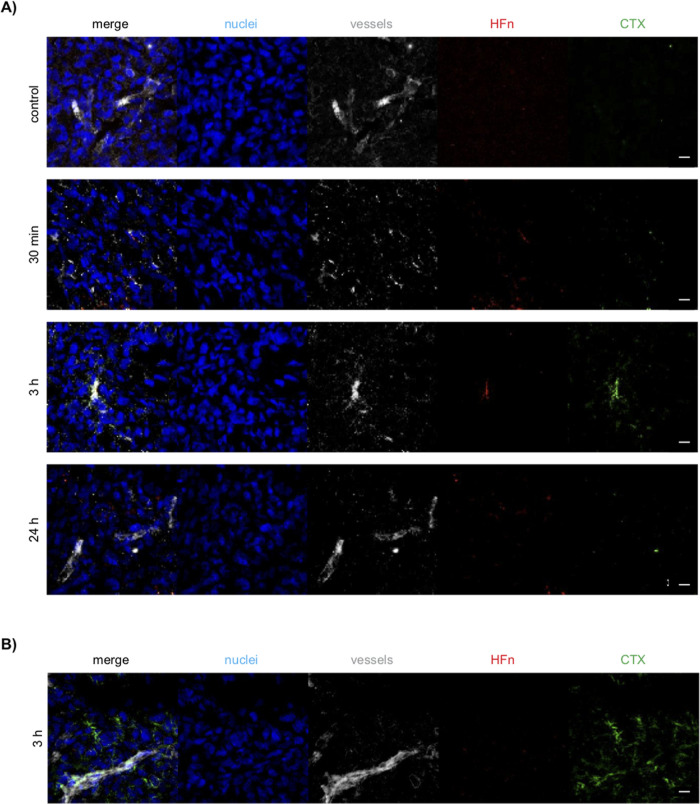
Intratumoral distribution of the HFn–CTX nanoconjugate or free CTX. (A) Representative images of 15 μm-thick cryosections of tumors excised 30 min, 1 h, 3 h, and 24 h after the injection of the nanoconjugate or PBS as a control (control). (B) Representative images of 15 μm-thick cryosections of tumors excised 3 h after the injection of unlabeled CTX. Confocal Z-stack images were acquired with a 0.5 μm step size. Stacks were summed together using ImageJ to obtain the images shown above. The background was subtracted using the rolling ball method with a radius of 4.0 pixels. Nuclei were stained with DAPI (blue), vessels with anti-CD31 antibody (gray), and CTX conjugated on the surface of HFn with anti-human AF488 antibody (green). AF647-labeled HFn is shown in red. Scale bar, 10 μm.

### Bioavailability of the HFn–CTX Nanoconjugate

Blood samples were collected from the retro-orbital plexus prior to injecting the HFn–CTX nanoconjugate and 30 min, 1 h, 3 h, and 24 h after, and the intensity of fluorescence was measured in these samples. The nanoconjugate signal progressively decreased over time (Figure S6A), in accordance with its biodistribution and clearance. We also determined the concentration of the HFn–CTX nanoconjugate using a calibration curve (Figure S6B) from which the percentage of injected dose (% of ID) in the plasma could be derived for each mouse. At 30 min post-injection, the plasma retained only about 38% of the ID (Figure 8A), showing that the HFn–CTX nanoconjugate readily reached both the organs and TNBC tumors (Figure S6C).

**Figure 8:**
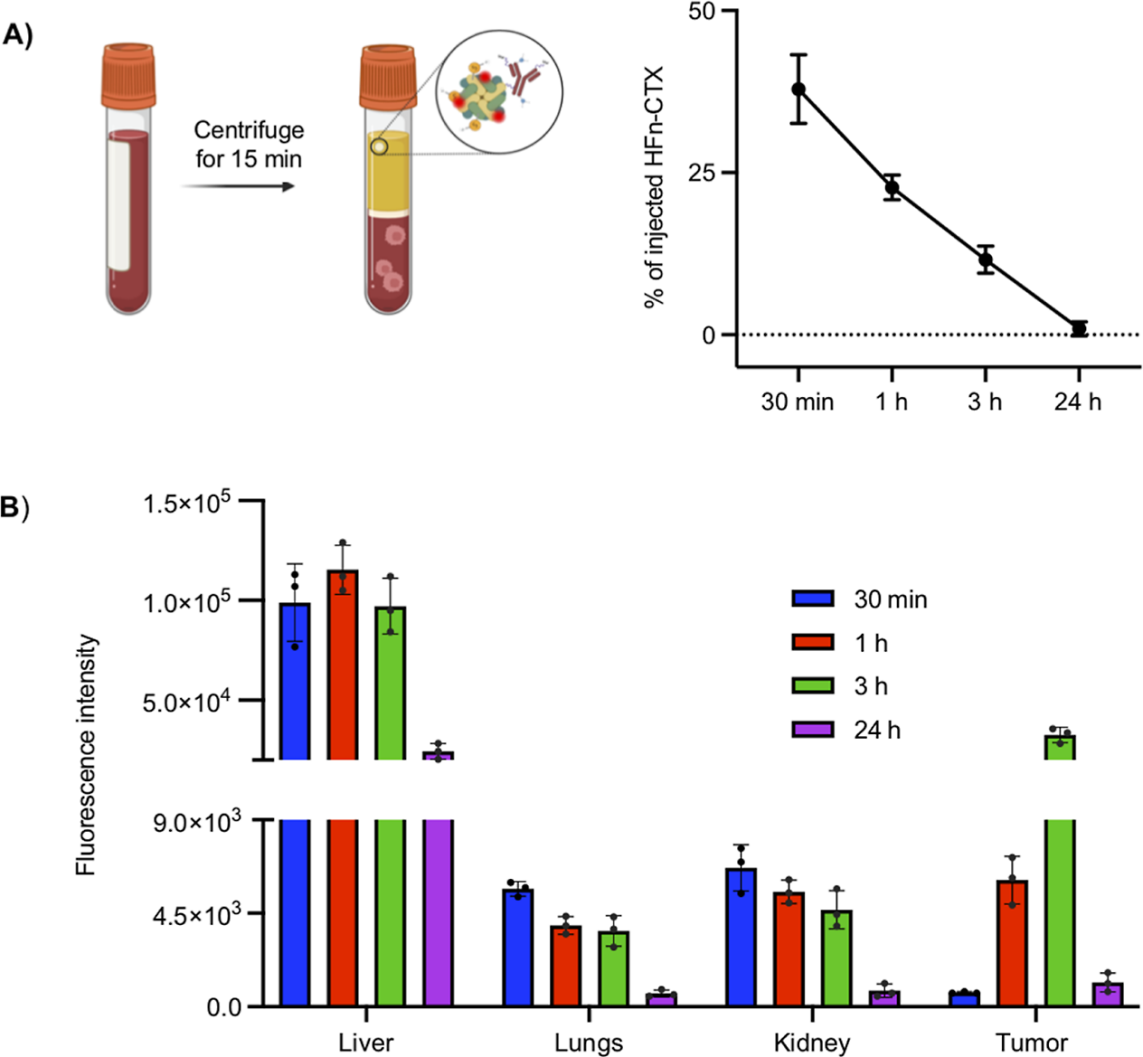
Distribution of the AF647-labeled HFn–CTX nanoconjugate in off-target organs and tumor homogenates. (A) Percentage of the injected dose (% of ID) of the AF647-labeled HFn–CTX nanoconjugate in mouse plasma. Reported values are mean ± s.e. of 3 different samples under each experimental condition. (B) Fluorescence intensity of the AF647-labeled HFn–CTX nanoconjugate measured in homogenated tumors and organs. Data represent mean ± s.e. of 3 different samples for each experimental condition.

To investigate the bioavailability of the nanoconjugate to the tumor and major organs, we exploited homogenized tissues (Figure 8B). We found that both the HFn–CTX nanoconjugate and free CTX accumulated preferentially in the liver (Figures 8B and S3B, respectively), as previously published for HFn alone and in agreement with the detoxification role of this organ.^52^ After 24 h, there was a consistent decrease in the fluorescence intensity in all organs tested (liver, kidneys, and lungs).

The tumor homogenates confirmed that the accumulation of the HFn–CTX nanoconjugate peaked at 3 h but revealed also a higher fluorescence signal already at 1 h post-injection, which was probably underestimated in the ex vivo analysis, due to its lower sensitivity. Confocal imaging of tumor cryosections corroborated this result: the HFn–CTX nanoconjugate was accumulated inside blood vessels at 1 and 3 h post-injection (Figure 7) and then presumably diffused across the tumor mass as it was no longer detectable at 24 h.

Importantly, the sum of these analyses showed that the tumor represents the second preferred site of accumulation and retention of the nanoconjugate, which thus appears to exhibit superior properties compared to HFn nanoparticles.^54,55^

## Discussion

CTX is an anti-EGFR monoclonal antibody that has proven potential in treating EGFR-positive cancers. However, primary or acquired mutations in the Ras-ERK and PI3K-AKT pathways confer resistance to CTX on most EGFR-positive tumors, such as glioblastoma and TNBC, for which limited molecular targets are available.

In this study, we developed a dual-targeting strategy that leverages HFn nanoconjugates to repurpose CTX for the treatment of CTX-resistant cancers. Our approach capitalizes on HFn to target the TfR1, which is highly expressed in nearly all solid tumors, and on the EGFR-binding ability and immune-mediated killing activity of CTX.

In this way, we enhanced the CTX’s antitumor efficacy on refractory cells: the ADCC response observed in TNBC spheroids treated with the HFn–CTX nanoconjugate was markedly stronger than that elicited by free CTX, suggesting that this dual-targeting approach effectively amplifies the immune-mediated antitumor response. Surprisingly, the nanoconjugate had no such effect on glioblastoma spheroids, revealing the existence of unappreciated factors that allow the nanoconjugate to boost the CTX’s immune-mediated antitumor response.

We identified the cell-surface levels of EGFR and TfR1 as key factors underlying this difference. The finding that the uptake of the nanoconjugate is faster in the U-87 MG cells than in the MDA-MB-231 cells is in line both with our observations and evidence from the literature, as summarized below.

First, U-87 MG cells show high levels of TfR1, which is known to undergo constitutive ligand-independent clathrin-mediated endocytosis (CME) and recycling.^49^ Second, they have a relatively low amount of EGFR on the plasma membrane. This is relevant because, at odds with TfR1, the endocytosis of EGFR is mainly driven by ligands that bind and activate the EGFR, thereby inducing its clathrin-dependent internalization. Third, ligand-free, inactive EGFR is predominant in most tumor microenvironments, where the concentration of EGFR ligands is usually low.^56^ Fourthly, CTX outcompetes EGFR ligands and could stimulate the uptake of ligand-free EGFR via alternative secondary routes such as caveolin-mediated endocytosis.^57,58^ Fifthly, caveolin-mediated endocytosis is also kinetically slower than CME, plays a negligible role in the uptake of nanoparticles, and might be negatively impacted by the nanoconjugate-mediated formation of TfR1-EGFR clusters.^59,60^ This collective evidence likely explains why the HFn–CTX nanoconjugate lingers longer on the plasma membrane in MDA-MD-231 than in U-87 MG cells and, thereby, triggers ADCC only in the former. Anyway, the ability of HFn–CTX to overcome resistance to CTX in TNBC underscores the power of targeting multiple receptors to improve therapeutic effectiveness and specificity.

In addition to enhancing ADCC in TNBC spheroids, the HFn–CTX nanoconjugate exhibited favorable biophysical properties, including a hydrodynamic size of <30 nm, compatible with tumor penetration. Indeed, it showed superior biodistribution and efficient accumulation in TNBC tumors in vivo compared to HFn, possibly resulting from the combined effects of size and dual targeting.^52^ Of note, the modular nature of the HFn nanocage allows for potential functionalization with other therapeutic mAbs or biologics, broadening its applicability beyond TNBC.

Although the therapeutic potential of the HFn–CTX nanoconjugate remains to be explored in full, we demonstrate the feasibility of repurposing CTX for TNBC therapy through a dual-targeting strategy that exploits HFn. Hence, the HFn–CTX nanoconjugate qualifies as a prime candidate for advanced nanomedicine applications to increase the clinical success of CTX-based therapies in TNBC and other refractory cancers.

## Conclusions

Our results demonstrate that dual targeting of EGFR and TfR1 via HFn–CTX nanoconjugates effectively enhances immune-mediated cytotoxicity in TNBC models. By prolonging CTX retention on the surface of EGFR-overexpressing tumor cells, the nanoconjugate triggers a robust ADCC response, even in CTX-resistant settings. Its favorable biodistribution and selective tumor accumulation further underscore the translational potential of this approach. In summary, our study lays the groundwork for repurposing CTX in TNBC and highlights the promise of HFn-based nanoplatforms for precision immunotherapy.

## Supplementary Material



## Notes

Physicochemical properties of HFn–CTX nanoconjugates; optimization of HFn labeling with AF488; optimization of CTX labeling with AF647; optimization of HFn labeling with AF647; optimization of CTX labeling with AF750; two-step purification of the HFn–CTX nanoconjugate by SEC–FPLC; distribution of the HFn–CTX nanoconjugate and HFn nanoparticles within GBM and TNBC spheroids; free CTX distribution in TNBC tumors and liver; large-field images of the uptake of the HFn–CTX nanoconjugate or HFn nanoparticles in U-87 MG and MDA-MB-231 cells; large-field images of cell-surface EGFR and TfR1 levels in U-87 MG and MDA-MB-231 cocultures; and biodistribution of the HFn–CTX nanoconjugate in mice (PDF)
